# Patients with mutations of the Thyroid hormone beta-receptor show an ADHD-like phenotype for performance monitoring: an electrophysiological study

**DOI:** 10.1016/j.nicl.2020.102250

**Published:** 2020-03-19

**Authors:** Jan Uter, Marcus Heldmann, Berenike Rogge, Martina Obst, Julia Steinhardt, Georg Brabant, Carla Moran, Krishna Chatterjee, Thomas F. Münte

**Affiliations:** a Department of Neurology, University of Lübeck, Lübeck, Germany; b Department of Psychology II, University of Lübeck, Lübeck, Germany; c Department of Internal Medicine I, University of Lübeck, Lübeck, Germany; d Department of Endocrinology, University of Cambridge, Cambridge, UK

**Keywords:** Thyroid hormones (TH), Resistance to thyroid hormones, TH beta receptor, Action monitoring, Event-related potentials, ADHD-like symptoms

## Abstract

Resistance to thyroid hormone beta (RTHβ) is a syndrome of reduced responsiveness of peripheral tissue to thyroid hormone, caused by mutations in the thyroid hormone receptor beta (THRB). Its cognitive phenotype has been reported to be similar to attention deficit hyperactivity disorder (ADHD). This study used electrophysiological biomarkers of performance monitoring in RTHβ to contribute further evidence on its phenotypical similarity to ADHD. Twenty-one participants with RTHβ aged 18–67 years and 21 matched healthy controls performed a modified flanker task during EEG recording. The RTHβ and control groups were compared on behavioural measures and components of event related potentials (ERPs), i.e. the error related negativity (ERN), the error positivity (Pe) and P3 component. There were no significant group differences with regard to behaviour. RTHβ subjects displayed significantly reduced ERN and Pe amplitudes compared to the controls in the response-locked ERPs. In addition, we observed reduced P3 amplitudes in both congruent and incongruent trials, as well as prolonged P3 latencies in RTHβ subjects in the stimulus-locked ERPs. Our findings reveal alterations in error detection and performance monitoring of RTHβ patients, likely indicating reduced error awareness. The electrophysiological phenotype of RTHß subjects with regard to action monitoring is indistinguishable from ADHD.

## Highlights

• Mutations in the thyroid hormone receptor beta (THRB) lead to relative hyperthyroidism in the brain.
• Electrophysiological biomarkers of performance monitoring (ERN and Pe components) show a pattern similar to ADHD in carriers of THRB mutations.
• The phenotype of THRB mutation carriers is indistinguishable from ADHD with regard to performance monitoring.

## 1 Introduction

Thyroid hormones (TH) have a strong modulatory effect on the development and function of the human brain (Bauer et al., 2008). This becomes apparent in terms of cognitive alterations frequently appearing in thyroid diseases. The effect of hypothyroidism on cognition can range from mild impairment in memory and attention to conditions resembling dementia or depression (Bauer et al., 2008). Symptoms like inattention, hyperarousal or cognitive function deficits, however, are more commonly associated with hyperthyroidism (Bauer et al., 2008; Göttlich et al., 2015).

Regarding resistance to thyroid hormone beta (RTHβ), insight about a distinct cognitive profile is scarce. RTHβ is a rare thyroid syndrome, which is caused by mutations in the gene encoding for the thyroid hormone receptor beta (THRB) (Dumitrescu and Refetoff, 2015; Refetoff et al., 2014). Characteristic findings in RTHβ patients are: elevated levels of TH, normal to slightly elevated levels of thyrotropin (TSH), goiter and the absence of usual symptoms of hyperthyroidism (Dumitrescu and Refetoff, 2015).

Previous studies revealed symptoms similar to attention deficit hyperactivity disorder (ADHD) (Hauser et al., 1993; McDonald et al., 1998; Stein et al., 1995; Weiss et al., 1993). In fact, ADHD was found to be a common manifestation of RTHβ, with around half of RTHβ patients developing ADHD (Dumitrescu and Refetoff, 2015). One of the hallmarks of typical ADHD are deficits in executive function and response control (Ehlis et al., 2018).

At the electrophysiological level, adults and children diagnosed with ADHD have shown alterations in amplitudes of event-related potential (ERP) components that are similarly indicative of executive dysfunction. Specifically, numerous studies have consistently reported reduced amplitudes of the error-related negativity (ERN) in ADHD patients (Ehlis et al., 2018; Lange-Malecki et al., 2018; Marquardt et al., 2018; Geburek et al., 2013; Herrmann et al., 2010; Liotti et al., 2005). The ERN is a fronto-central negative deflection that occurs 0–100 milliseconds (ms) after an erroneous response in a choice task (Falkenstein et al., 2000, 1991; Gehring et al., 1993) in the ERP time-locked to the subject's response. The ERN has been identified as a marker of performance monitoring, either viewed as reflecting error-detection proper (Falkenstein et al., 1995; Gehring et al., 1993), the degree of response conflict experienced by the subject (Botvinick et al., 2001), or a reflection of an internal comparison of two signals: an unconscious representation of the ongoing action and a conscious representation of the intended one (Dehaene, 2018). Concerning the origin of the ERN, magnetic resonance imaging (MRI) as well as EEG source localization, have pointed to the anterior cingulate cortex (ACC) (Botvinick et al., 2004; Herrmann et al., 2004; van Veen and Carter, 2002). Following the ERN, response-locked ERPs are characterized by the error positivity (Pe) which has been linked to error awareness (Falkenstein et al., 2000; Ullsperger et al., 2010). For example, Steinhauser and Yeung (2012) suggested that the Pe reflects accumulated evidence for an error which in turn drives the emergence of error awareness. Adults with ADHD have been reported to have reduced Pe amplitude (Marquardt et al., 2018; Balogh et al., 2017, Wiersema et al., 2009) suggesting decreased error awareness in this group.

With regard to stimulus-locked ERPs, reduced P3 amplitudes have been reported in ADHD (Kim et al., 2014; Szuromi et al., 2011; Wiersema et al., 2009). This reduction has been suggested to be an endophenotype for ADHD. In a meta-analysis of 6 studies Szuromi et al. (2011) found a consistent reduction of P3 to targets in adult ADHD patients with a moderate effect size. Preceding the P3 in time, a frontocentral negativity (“N2”) has been described that is usually greater for incongruent flanker stimuli compared congruent stimuli (van Veen and Carter, 2002; Riba et al., 2005) and thought to reflect the conflict induced by the incongruous stimuli. To the best of our knowledge, this component has not been described to be altered in ADHD.

Against this background, we used the ERN, Pe, N2, and P3 components of the ERP to investigate the electrophysiological signature of performance monitoring of RTHβ. Taking the results of ADHD studies into account, we hypothesised that RTHβ patients would display reduced ERN, Pe, and P3 amplitudes, suggesting deficits in performance monitoring, error awareness and target processing. We had no hypothesis for the N2, as this component has not been reported to be altered in ADHD.

## 2 Materials and methods

### 2.1 Ethic statement

The Ethics Committee of the University of Lübeck approved all procedures prior to the experiment. All subjects gave their written informed consent prior to participation.

### 2.2 Participants

**Fig. 1:**
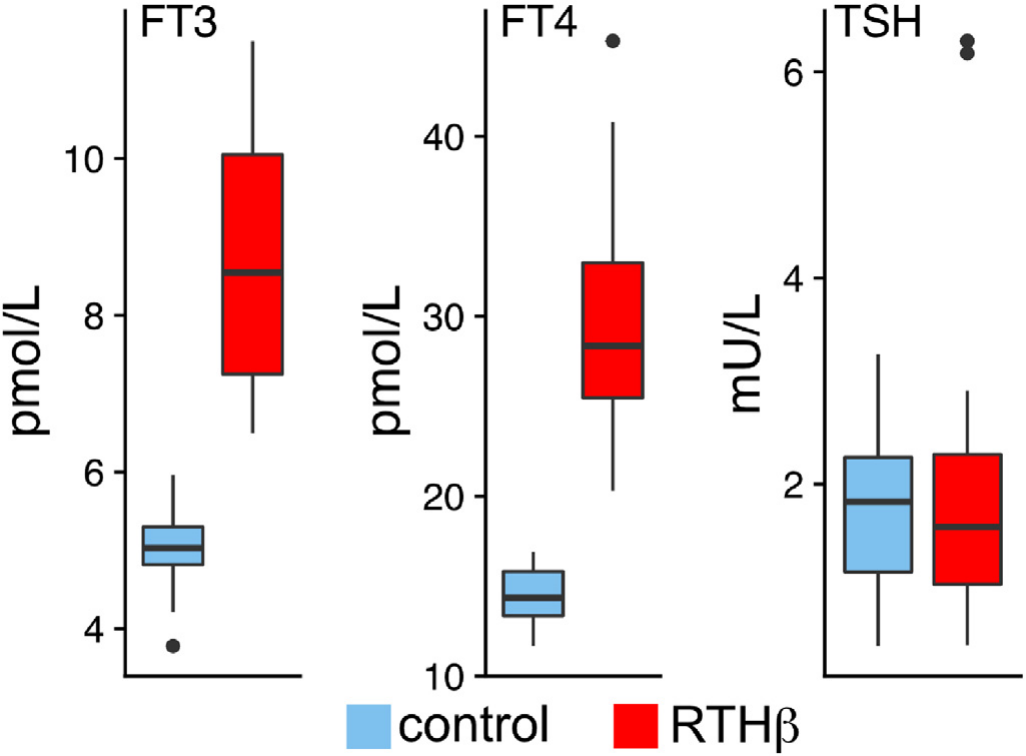
Mean FT3, FT4, and TSH levels per group. Box plots display interquartile range (IQR, 25th–75th percentile) and the 50th percentile of the data. Whiskers indicate 1.5 x IQR.

This study was part of a collaboration between the University of Cambridge in the United Kingdom (UK) and the University of Lübeck in Germany. Twenty-one adults from the UK diagnosed with RTHβ (mean age 39 y, SD 15.0, 12 women) were matched with 21 healthy adults (mean age 38 y, SD 14, 12 women). Regarding the educational degree of each group, eight reached O-level and 13 A-level. The A-level (Advanced Level) is the highest school qualification in the UK and is generally required for university entrance. The O-level (Ordinary Level) qualification is the secondary school-leaving qualification in the UK. Further qualification levels such as university degrees or formal education were also incorporated in the matching process. χ^2^ tests revealed no significant differences regarding sex or educational level between groups (all χ^2^ < 0.0012, all *p* > 0.6), a two sample t-test showed no significant age differences (t(36) = 0.02, *p* > 0.95). Both groups were tested in the research facilities of the University of Lübeck (UKSH). A neurologist with additional training in psychiatry (author TFM) examined all 42 participants prior to the study for their general health, as well as past and current neurological and psychiatric conditions. Besides subjective complaints of being inattentive, made by some members of the RTHβ group, no further conditions emerged. Furthermore, a neuroradiologist assessed MRI images, which all yielded no structural abnormalities. Additionally, blood samples regarding TH status, were collected and analysed. The laboratory analysis of TSH, fT3, fT4 was performed in Cambridge, using enzyme-linked immunosorbent assay (EILSA). The standard values of hormone levels were as follows: fT3 3.5–6.5 pmol/l, fT4 10–19.8 pmol/l and TSH 0.35–5.5 mU/l. Members of the RTHβ group had normal levels of TSH, except for two, who had slightly increased TSH. All of them had elevated levels of fT3 and fT4. All control subjects had TH and TSH levels inside the reference range (Fig. 1). THRB mutations of the RTHβ group were as follows: R320H (*n* = 5), R438H (4), R429Q (3), R383C (2), M310V (1), G345C (1), P453S (1), R243W (1), T227I (1), R338W (1), E460K (1). Due to massive artefacts during EEG-recording four participants were excluded from the analysis resulting in a final sample of 20 RTHβ patients and 18 healthy controls.

### 2.3 ADHD assessment

The Adult ADHD Self-Report Scale (ASRS-v1.1) and the ADHD Rating Scale-IV were used to assess self-reported symptoms of ADHD. The ADHD rating scale IV comprises two subscales, inattention and hyperactivity-impulsivity, and is based on 18 items in a 4 point Like scale format. The scale has shown good internal consistency, good test-retest reliability and validity as it predicts diagnostic status, as well as classroom behaviour, task accuracy and has been shown to be sensitive to treatment effects (e.g., DuPaul et al. 1998; Burns et al., 2013; Vallejo-Valdivielso et al., 2019). The ASRS has been developed by the WHO in conjunction with revision of the WHO Composite International Diagnostic Interview (CIDI). It has been validated as a diagnostic tool for assessing current ADHD symptoms in adults 18 years or older (Kessler et al., 2005, Kessler et al., 2005, 2007; Kiatrungrit et al., 2017).

Compared to controls, the RTHβ group showed significantly higher test results on both scales (ASRS-v1.1: control 60.9 (23.7), RTHβ 92.9 (40), t(36) = −2.9, *p* < 0.005; AHDH Rating Scale-IV: control 23.7 (7.6), 40.2 (9.5), t(36) = −4.17, *p* = 0.001).

### 2.4 Paradigm

The paradigm was a modified version of the Eriksen flanker task (Eriksen and Eriksen, 1974). In our variant of the task, test-subjects were briefly presented with an array of five arrows, with a central arrow serving as the target stimulus and two flanking arrows above and below the target stimulus. The flanking arrows pointed either to the same direction as the target stimulus (congruent), or to the opposite one (incongruent). Target and flanker stimuli were presented simultaneously. Stimuli were displayed in white colour on a black background, stimulus size was 7° by 15° visual angle in height and width. Subjects were instructed to fixate either to the fixation dot presented in the absence of a stimulus or to the target stimulus. It was the subjects’ task to respond as fast and as accurate as possible by pressing the button with their left index finger, if the arrow pointed to the left side and to press the right index finger in case the target pointed to the left side. Required button presses were equally distributed to the left and right index finger. We presented 480 incongruent and 320 congruent trials in 10 blocks of 80 trials (see Rodriguez-Fornells et al., 2002). Each flanker stimulus was presented for 100 ms; ISI varied between 900 and 1100 ms and was equally distributed within each block. Responses were captured using two Razor Abyssus gaming mice running with a 1 KHz polling (sampling) rate.

### 2.5 Electrophysiological methods

The EEG was recorded with 16 active dry electrodes (Sahara Active Dry Electrode System, g.tec medical engineering GmbH), scalp electrode positions: F3, Fz, F4, Fc5, Fcz, Fc6, C3, Cz, C4, Cp5, Cp6, P3, Pz, P4, Oz and A2, mounted in an elastic cap, referenced against an electrode placed at the left mastoid and a ground electrode at approximately AFz. To measure horizontal and vertical eye movements (VEOG, HEOG) two electrodes were placed lateral to the left and right eye's external canthus and to the supra- and infraorbital ridge of the left orbit. Since a dry electrodes system was used, impedances could not be measured. The frequency range that can be captured with this system is limited to 0.1–40 Hz. In order to account for amplitude differences, epoched EEGs were individually normalized using z-transformation. EEG and EOG were digitized with a sampling rate of 256 Hz.

EEG data were pre-processed using EEGlab (Delorme and Makeig, 2004) and ERPlab (Lopez-Calderon and Luck, 2014) toolboxes, running under Matlab 2017b (MATLAB and Statistics Toolbox Release 2017b, The MathWorks, Inc., Natick, Massachusetts, United States). First, data were filtered, using 0.5 Hz high-pass and 30 Hz low-pass filters. Next, data were offline re-referenced to the mean activity of the two mastoid electrodes. Stimulus-locked and response locked-data were epoched separately with an epoch length of 3000 ms (−1500 to +1500 ms to stimulus / response onset). Response locked data were categorized into correct and erroneous response bins, stimulus locked data into bins defined by congruent and incongruent stimulus followed by a correct response. To control for artifacts, epoched data were visually inspected and epochs containing non-EOG artifacts were excluded from further analysis. In order to correct for EOG artefacts the remaining data were subjected to an independent component analysis (ICA) comprising all EEG and EOG channels. In the present study the infomax ICA algorithm (Bell and Sejnowski, 1995) implemented in EEGLAB was used. Components containing EOG activity were identified by visual inspection and removed from the EEG activity by subtracting these EOG components (see Delorme and Makeig, 2004). ERPs were calculated by averaging corresponding trials per subject and condition with a baseline of −300 to 0 ms for the response locked and −100 to 0 for the stimulus locked ERPs. Then, ERPs were filtered applying a 1–8 Hz bandpass filter to the response locked and 20 Hz lowpass filter to the stimulus locked data. Finally, the averages of all subjects were collapsed to calculate the grand average. To parameterize the response locked ERN component the mean amplitude 0–100 ms at the electrode position Fz, Fcz, Cz and Pz was calculated per subject. The number of trials used for calculating individual response locked ERPs was for the error condition 56.4 (congruent = 11.99, incongruent = 42.75) in the RTHβ and 44.16 (congruent = 11.33, incongruent = 32.83) in the control group, for correct responses the number of trials was 527.07 (congruent = 228.30, incongruent = 298.75) in the RTHβ and 668.88 (congruent = 273.11, incongruent = 295.77) in the control group. The subsequent PE component was parameterized at the same midline electrodes by the mean amplitude between 250 and 450 ms after button press (Falkenstein et al., 2000).

Latency differences of P3 amplitude were quantified by identifying the peak amplitude between 200 and 500 ms at the electrode positons Fz, Fcz, Cz, and Pz using the measurement routine implemented in ERPLAB. This analysis was done on the unfiltered data. Mean amplitudes were calculated by averaging the amplitude in a time window ±50 ms to the mean peak latency of each electrode and condition.

### 2.6 Behavioural data

Reaction time effects were determined by calculating the mean reaction time per subject and condition resulting in four different conditions: congruent correct, incongruent correct, congruent error, and incorrect error. To determine post-error slowing (PES) we identified response sequences where the last two responses before and the first response following an erroneous response were correct responses. PES was calculated by subtracting the correct response's reaction time before an error from the correct response's reaction time following an error.

### 2.7 Statistics

All analyses were performed with the ezANOVA (v4.4) package running under R 3.5.1. ANOVAs for ERN amplitude, N2 amplitude, P3 latency and P3 amplitude were three-way mixed models comprising the between subjects factor group (control/RTHβ) and the within subjects factors electrodes (Fz, Fcz, Cz, and Pz) and condition (ERN amplitude: error/correct, N2 amplitude congruent correct / incongruent correct, P3 amplitude and latency: congruent correct/incongruent correct). Initially, we calculated for the response locked ERN amplitude an ANOVA containing a congruency (congruent/incongruent) and a performance (error/correct) factor. Since the congruency failed to become significant we collapsed the data across the performance condition in order to increase the signal to noise ratio. To ensure that potential error-related group differences are not driven by signal-to-noise ratio effects we calculated an additional ANOVA for the error related response locked ERPs only (between-subjects-effect group (factor levels: RTHβ, control), within-subjects-effects electrode (Fz, Fcz, Cz, Pz), congruency (congruent, incongruent)).

Reaction times were also analysed with a three-way mixed model ANOVA and the between subjects factor group and the within subjects factor congruency (congruent, incongruent) and performance (correct, error). Post error slowing was analysed with a two-way mixed model ANOVA group and congruency. To correct for violation of sphericity Greenhouse-Geisser correction was applied. We are reporting uncorrected degrees of freedom, but corrected p-values. To test for group effects in post-error slowing we used a two sample t-test.

## 3 Results

### 3.1 Behavioural results

**Fig. 2:**
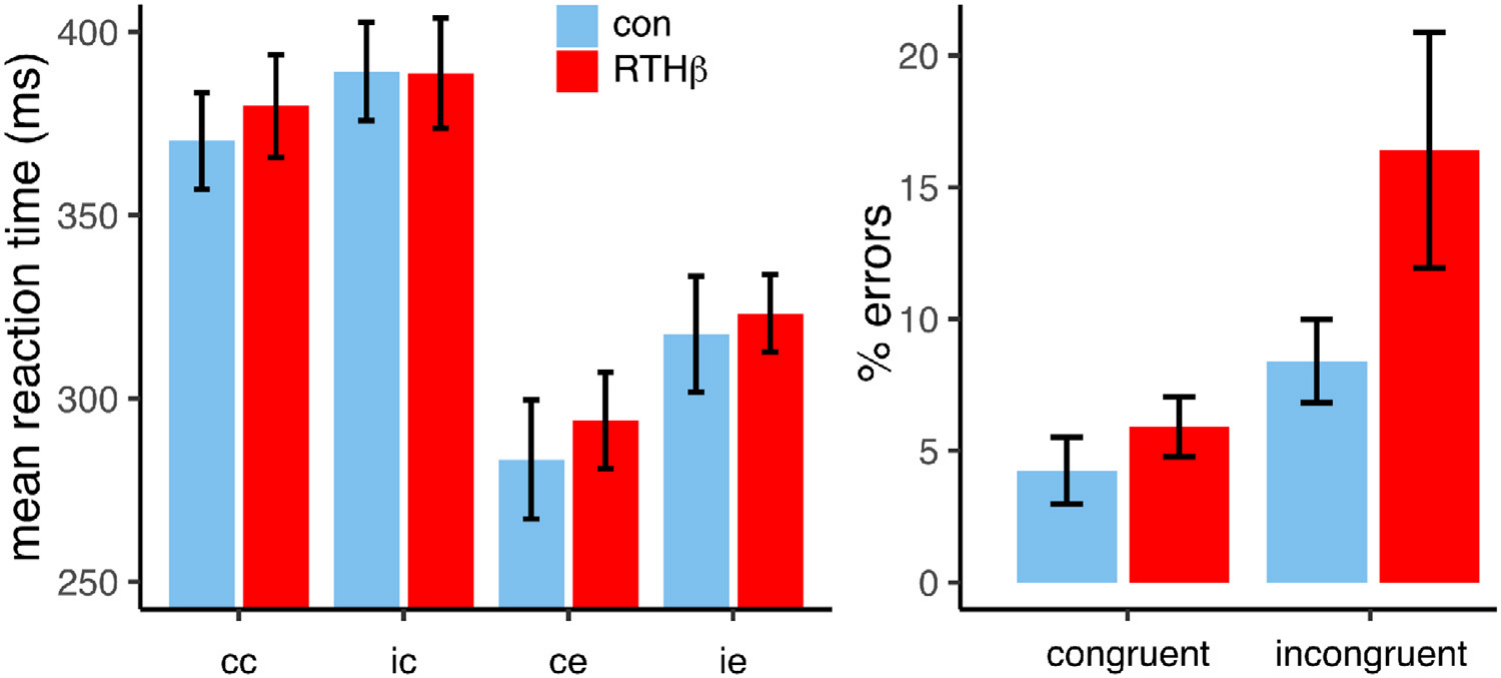
Mean reaction times and mean percent errors per group and condition. cc = congruent correct, ic = incongruent correct, ce = congruent error, ie = incongruent error.

Reaction times were significantly slower for correct than for erroneous responses (Fig. 2), (F (1,36) = 154.7, p<0.001, η^2^_g_ = 0.01). Furthermore, reaction times to incongruent stimuli were longer (F (1,36) = 21.32, *p* < 0.001, η^2^_g_ = 0.04). Erroneous responses to an incongruent stimulus were slower leading to a congruency by correctness interaction (F (1,36) = 7.15, *p* = 0.01, η^2^_g_ = 0.01). There was neither a group main effect nor an interaction of group with the other factors for reaction time (all F(1,36) < 0.57, n.s.).

All subjects made more errors in incongruent trials (Fig. 2) (F (1,36) = 12.5, *p =* 0.001, η^2^_g_ = 0.1). but this effect was more pronounced in the RTHß group, reflected by a trend towards a group x condition interaction group by congruency interaction (F (1,36) = 2.34, *p =* 0.13, η^2^_g_ = 0.02). There was no significant group main effect (F (1,36) = 2.49, *p =* 0.11).

Post-error slowing amounted to 42.7 ms (SD 44.2) in the RTHß group and 44.8 ms (SD 58.1) in the controls (t(36) = −0.12, *p =* 0.9).

### 3.2 ERPs: response-locked data

**Fig. 3:**
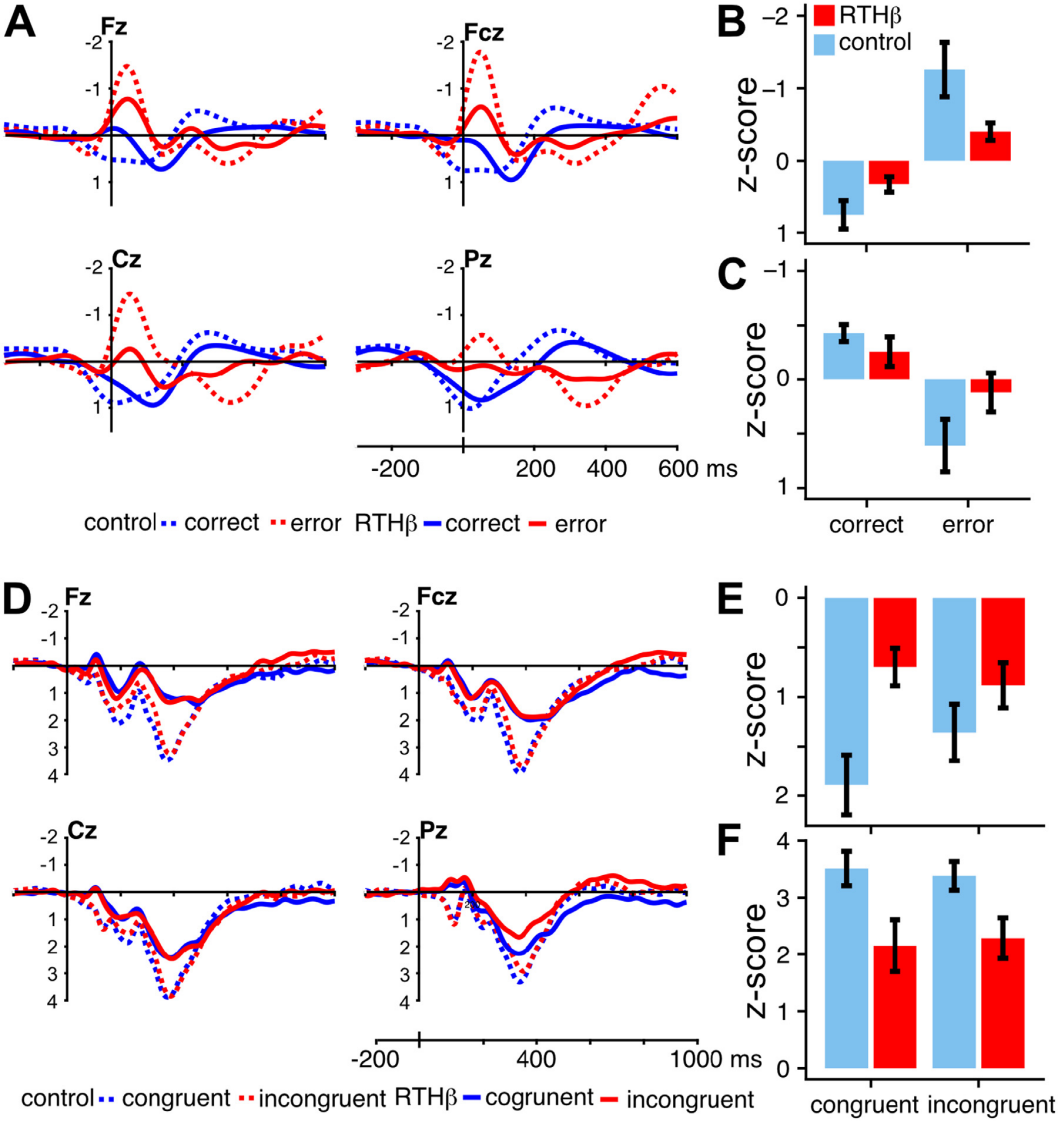
A: /-transformed response locked ERPs. B: mean z-scores of the ERN effect at Fcz. C: mean z-scores of the PE-effect at Cz. D: z-transformed stimulus locked ERPs. E: mean z-scores of the N2 effect at Fz. F: mean z-scores of P3 effect at Cz.

Fig. 3A,B highlights that correct and erroneous answers were differentiated by a robust ERN to the incorrect answers (main effect condition: F (1,36) =  46.05, *p* < 0.001, η^2^_g_ =  0.33) and that the RTHβ group had considerably smaller ERN amplitudes (group by condition, F (1,36) =  7.52, *p* =  0.009, η^2^_g_ = 0.07). An interaction condition by electrodes was also present (F (3,108) = 3.44, *p* < 0.027, η^2^_g_ = 0.01), indicating larger ERNs for frontal and central electrodes. A subsequent ANOVA comprising erroneous trials only revealed a significant group (F (1,36) =4.28, *p =* 0.048, η^2^_g_ = 0.2) and electrode (F(3,108)=10.14, p<0.001, η^2^_g_ = 0.05), but not a significant congruency main effect (F(1,36)= 0.01, p<0.001, η^2^_g_ < 0.01). Moreover, no interaction became significant in this model (all *p >* 0.26). Because of the considerable age range of the participants and the known effects of age on the ERN amplitude (e.g., Niessen et al., 2017), an additional ANOVA for the ERN amplitude (errors only) was calculated using age as between subjects covariate. With this covariate the group effect remained significant (F(1,35)= 4.17, *p =* 0.049). Following the ERN, a typical Pe component was observed (Fig. 3A,C), which was larger for the error trials (main effect condition: F (1,36) = 34.44, *p* < 0.001, η^2^_g_ = 0.26). Moreover, the Pe effect, i.e. the difference between correct and error trials was smaller in the RTHß group, as reflected by a group x condition interaction (F (1,36) =4.20, *p* = 0.048, η^2^_g_ = 0.04). There was no effect of group for the Pe (F (1,36) = 1.04, *n.s.*).

### 3.3 ERPs: stimulus-locked data

The stimulus-locked data are characterized by a typical succession of ERP components. Following the first negativity N1, peaking at about 100 ms, a frontal second negativity N2 with a peak latency of about 280 ms can be observed, which is followed by a positive deflection peaking around 400 ms (P3 component, Fig. 3D,F). Differential effects were first observed for the N2 that was quantified at the Fz electrode (mean amplitude in the 200–400 ms time-window) following earlier investigations (van Veen and Carter, 2002; Riba et al., 2005). This component showed a congruency effect in the controls but not in the RTHß group (group x condition interaction, F (1,36) = 8.32, *p* < 0.007, η^2^_g_ = 0.03). Also, a group effect was observed (F (1,36) = 6.24, *p* = 0.017, η^2^_g_ = 0.13). To rule out that the N2 effect was driven by an overlapping slow positivity, the analysis was repeated after filtering the data with a bandpass of 4–12 Hz (see Luu and Tucker, 2001, for a similar approach). The group and condition main effects for the N2 remained statistically significant with no group x condition interaction.

**Fig. 4:**
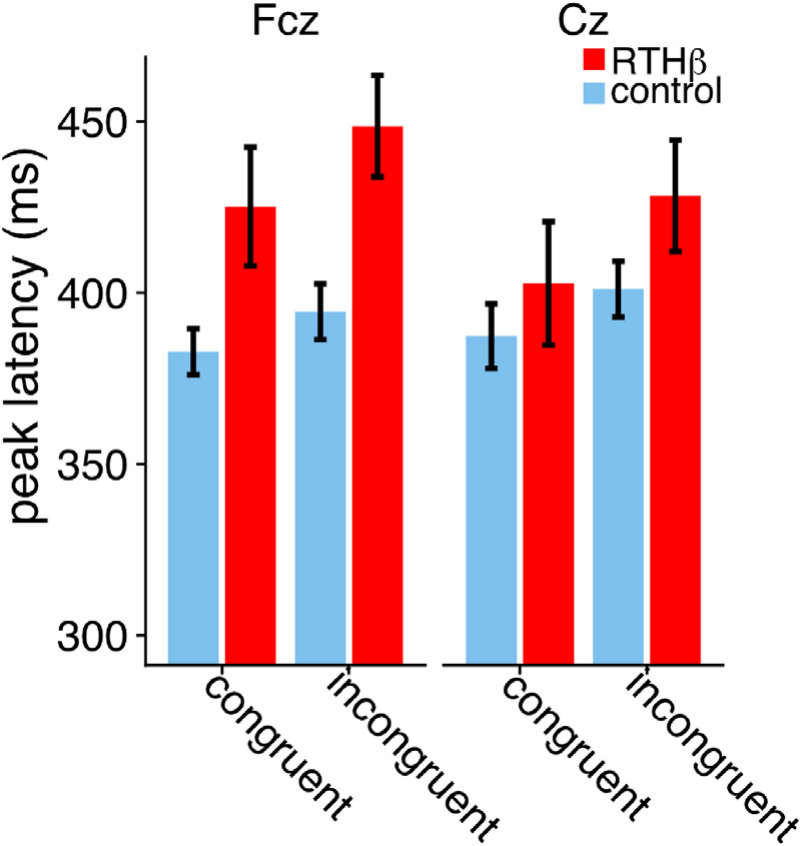
Mean latencies of P3 component.

The P3 amplitude was significantly smaller in the RTHβ group, reflected by a main effect of group (F (1,36) = 22.94, *p* < 0.001, η^2^_g_ = 0.22). In addition, amplitudes were larger at fronto-central electrode sites, indicated by a main effect of electrode (F (1,36)= 5.17, *p* = 0.004, η^2^_g_ = 0.05). We did not observe a significant difference in amplitude between congruent and incongruent trials for both groups (F (1,36) = 1.09, n.s.). Besides that, we found that RTHβ subjects had delayed P3 peak latencies compared to controls, which was demonstrated by a main effect of group (F (1,36) = 4.17, *p* < 0.049, η^2^_g_ = 0.07). P3 peaked later for incongruent stimuli, illustrated by a main effect of condition (F (1,36) = 8.09, *p* < 0.007, η^2^_g_ = 0.01, Fig. 4). Neither the electrode main effect nor any interaction became significant (all *p >* 0.14).

## 4 Discussion

This study investigated the electrophysiological markers of performance monitoring in RTHβ patients using a flanker task paradigm. Because of previous indications of an ADHD-like phenotype (Dumitrescu and Refetoff, 2015; Hauser et al., 1993; McDonald et al., 1998; Stein et al., 1995; Weiss et al., 1993), we hypothesized that RTHß subjects would exhibit a similar pattern of behavioural and electrophysiological effects as ADHD patients, i.e. increased errors and reduced amplitudes of the ERN, Pe and P3 components of the ERP (for a meta-analysis demonstrating the generality of these effects: Geburek et al., 2013).

This prediction was borne out, albeit only for the ERP components and not for the behaviour. Electrophysiologically, we did not predict but nevertheless found an absent congruency effect on the N2 component in the RTHß group in the stimulus-locked ERP (Van Veen and Carter, 2002; Rey-Mermet et al., 2019; Riba et al., 2005).

In addition, an ADHD-like phenotype in the RTHß subjects was further corroborated by the significantly higher scores of RTHβ subjects on ADHD assessment scales.

We will now discuss the different effects. While both groups, RTHß and controls, made more errors in incongruent trials than in congruent ones, the apparent increased error rate for incongruent trials in RTHß was not significant and therefore will not be discussed further. There was no difference in reaction times between the groups which has been found in some (Bluschke et al., 2016; van Meel et al., 2007) but not all (Geburek et al., 2013; Herrmann et al., 2010) ADHD studies. Post error slowing (PES), a post-error-adaptation effect, where subjects have slower RT after an incorrect answer (Danielmeier and Ullsperger, 2011) was present, but virtually identical for both groups.

The ERN component has been established as a marker of performance monitoring and has been found to be attenuated in ADHD in most studies (see Geburek et al., 2013, for a meta-analysis) with only a few exceptions (Groom et al., 2010; Shiels and Hawk, 2010; Wiersema et al., 2009). The reduction of the ERN component in the current study suggests therefore a similarity to ADHD.

Likewise, the Pe component has been found to be reduced in ADHD (Marquardt et al., 2018; Balogh et al., 2017, Wiersema et al., 2009) and it has also been found reduced in the current study in the RTHß group. The most prevalent interpretation of the Pe has been that it correlates with error awareness (Falkenstein et al., 2000). Thus, the reduced amplitude of the Pe would indicate a decreased error awareness in the RTHß participants. More specifically, the account of Steinhauser and Yeung (2010) posits that it reflects the accumulated evidence that an error has been committed. With regard to the ERN, Wessel (2012) has suggested that it “serves as a feed-forward input signal into the systems responsible for error awareness. Alongside the input from many other areas in which error-relevant information is coded, the ultimate emergence of ‘error awareness’ is grounded on the amplitude of this input.” Taken together, this suggests that the RTHß subjects have a problem with error processing, most likely with the conscious appreciation of errors. This hypothesis should be followed up, for example using a paradigm requiring the signalling of a self-detected error by the subject (Wessel, 2012) or by adding autonomous nervous system measures (skin conductance response, heart rate changes) which are highly sensitive to subjective error awareness (O'Connell et al. 2007; Wessel et al. 2011). Moreover, as the current study did not employ an ADHD group but rather relied on previous studies in adult ADHD participants as well as on a meta-analysis (Geburek et al., 2013), follow-up studies might benefit from the inclusion of an additional control group of ADHD patients.

The stimulus-locked data revealed significantly reduced P3 amplitudes and prolonged P3 latencies in RTHβ subjects, compared to the controls. P3 amplitude is associated with attentional resource allocation and is influenced by cognitive demands during task processing (Polich, 2007). We observed an attenuation of P3 amplitude in RTHβ subjects, regardless of stimulus condition, which suggests deficits in attentional processes again similar to previous studies in ADHD (Fisher et al., 2013; Marquardt et al., 2018; McLoughlin et al., 2009; Szuromi et al., 2011).

Finally, a modulation of the N2 component in the stimulus-locked ERP as a function of congruency (van Veen and Carter, 2002; Riba et al., 2005; Krämer et al., 2007) was present in the control participants but not in the RTHß group, suggesting a diminished sensitivity of this group to stimulus conflict. This effect has not been investigated thoroughly with regard to ADHD. Therefore, it should be followed up by additional experiments.

### 4.1 Conclusion

We have established an electrophysiological phenotype of RTHß that is virtually indistinguishable from that found in ADHD, thus confirming and extending earlier more informal observations. This phenotype should be further specified, i.e. by the implementation of dual task and task switching paradigms taxing the cognitive system to a greater extent (Bueno et al., 2017; Roberts et al., 2012; King et al., 2007). Moreover, better understanding of the cognitive phenotype of RTHß will also lead to improved treatment, be it pharmacological (Groom et al., 2013) or behavioural (Schoenberg et al., 2014).

## CRediT authorship contribution statement

**Jan Uter:** Formal analysis, Writing - original draft. **Marcus Heldmann:** Investigation, Visualization, Formal analysis, Writing - review & editing. **Berenike Rogge:** Investigation, Writing - review & editing. **Martina Obst:** Investigation, Writing - review & editing. **Julia Steinhardt:** Investigation, Writing - review & editing. **Georg Brabant:** Conceptualization, Resources, Writing - review & editing, Funding acquisition. **Carla Moran:** Conceptualization, Resources, Writing - review & editing. **Krishna Chatterjee:** Conceptualization, Resources, Writing - review & editing. **Thomas F. Münte:** Conceptualization, Resources, Writing - original draft, Supervision, Funding acquisition.
